# 1,3-Bis(3-phenyl­prop­yl)-1*H-*benz­imidazole-2(3*H*)-tellurone

**DOI:** 10.1107/S1600536808004893

**Published:** 2008-02-22

**Authors:** Şerife Pınar Yalçın, Mehmet Akkurt, Ülkü Yılmaz, Hasan Küçükbay, Orhan Büyükgüngör

**Affiliations:** a Department of Physics, Faculty of Arts and Sciences, Erciyes University, 38039 Kayseri, Turkey; b Department of Chemistry, Faculty of Arts and Sciences, Ínönü University, 44280 Malatya, Turkey; c Department of Physics, Faculty of Arts and Sciences, Ondokuz Mayıs University, 55139 Samsun, Turkey

## Abstract

The title compound, C_25_H_26_N_2_Te, was synthesized from bis­[1,3-bis­(3-phenyl­prop­yl)benzimidazolidin-2-yl­idene] and Te in a toluene solution. The molecule possesses a twofold rotation axis passing through the Te atom and the center of the benzimidazole ring system. The benzimidazole ring system makes an angle of 67.9 (4)° with the phenyl rings.

## Related literature

For related literature, see: Akkurt *et al.* (2004*a* ▶,*b* ▶, 2005 ▶); Aydın *et al.* (1999 ▶); Chakravorty *et al.* (1985 ▶); Karaca *et al.* (2005 ▶); Lappert (1988 ▶); Lappert *et al.* (1980 ▶); Roeterdink *et al.* (1983 ▶); Türktekin *et al.* (2004 ▶); İngeç *et al.* (1999 ▶); Närhi *et al.* (2004 ▶); Sadekov *et al.* (1998 ▶); Singh *et al.* (2006 ▶).
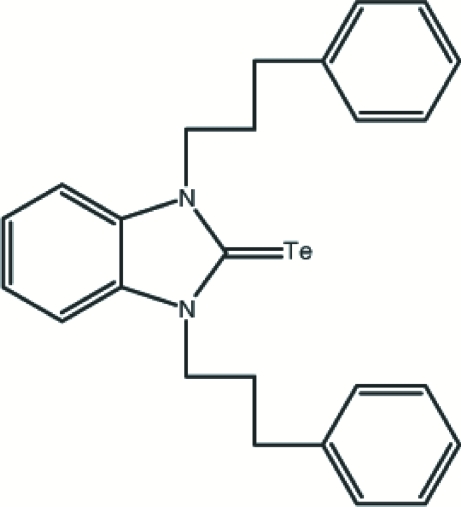


## Experimental

### 

#### Crystal data

C_25_H_26_N_2_Te
*M* *_r_* = 482.08
Tetragonal, 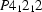

*a* = 10.6004 (2) Å
*c* = 20.4365 (6) Å
*V* = 2296.42 (9) Å^3^
*Z* = 4
Mo *K*α radiation
μ = 1.31 mm^−1^
*T* = 296 K
0.54 × 0.48 × 0.39 mm

#### Data collection

Stoe IPDSII diffractometer
Absorption correction: integration (*X-RED32*; Stoe & Cie, 2002 ▶) *T*_min_ = 0.539, *T*_max_ = 0.630
28301 measured reflections
2264 independent reflections
2166 reflections with *I* > 2σ(*I*)
*R*_int_ = 0.049

#### Refinement

*R*[*F* ^2^ > 2σ(*F* ^2^)] = 0.037
*wR*(*F* ^2^) = 0.105
*S* = 1.09
2264 reflections
99 parameters
3 restraints
H-atom parameters constrained
Δρ_max_ = 0.92 e Å^−3^
Δρ_min_ = −0.48 e Å^−3^
Absolute structure: Flack (1983 ▶), 889 Friedel pairs
Flack parameter: 0.01 (6)

### 

Data collection: *X-AREA* (Stoe & Cie, 2002 ▶); cell refinement: *X-AREA*; data reduction: *X-RED32* (Stoe & Cie, 2002 ▶); program(s) used to solve structure: *SIR97* (Altomare *et al.*, 1999 ▶); program(s) used to refine structure: *SHELXL97* (Sheldrick, 2008 ▶); molecular graphics: *ORTEP-3 for Windows* (Farrugia, 1997 ▶); software used to prepare material for publication: *WinGX* (Farrugia, 1999 ▶).

## Supplementary Material

Crystal structure: contains datablocks global, I. DOI: 10.1107/S1600536808004893/ez2117sup1.cif

Structure factors: contains datablocks I. DOI: 10.1107/S1600536808004893/ez2117Isup2.hkl

Additional supplementary materials:  crystallographic information; 3D view; checkCIF report

## Figures and Tables

**Table 1:** Selected bond lengths (Å)

Te1—C1	2.058 (4)

**Fig. 1.**
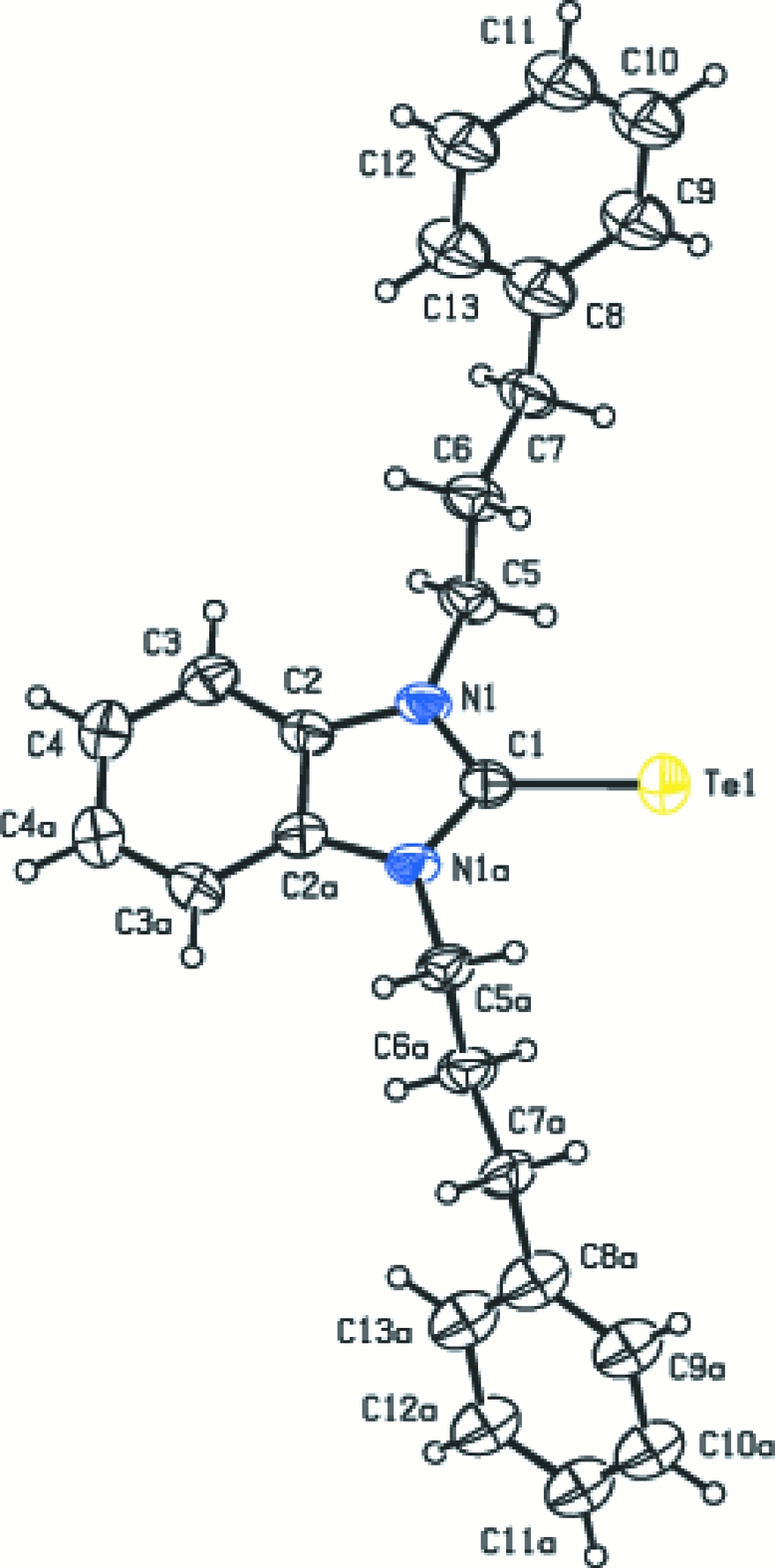
The molecular structure of the title compound (I) with the atom numbering scheme. Displacement ellipsoids for non-H atoms are drawn at the 30% probability level. [Symmetry code: (a) -y + 1, -x + 1, -z + 3/2.]

**Fig. 2.**
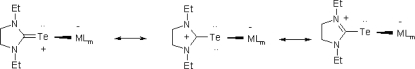
The resonance hybrids of tellurourea metal complexes (Lappert et al., 1980).

## supplementary crystallographic information

### Comment

Electron-rich olefins are powerful reducing agents (Lappert, 1988). It is known that the ultimate oxidation products of electron-rich olefins with air are ureas; sulfur and selenium react similarly to form the corresponding analogues (Roeterdink *et al.*, 1983; Lappert *et al.*, 1980). The conversion of an electron-rich olefin into a tellurourea has a parallel in these known olefin reactions (Lappert *et al.*, 1980). There are extensive studies of cyclic ureas containing imidazolidine groups, including their X-ray crystal structures. However, there is no example of an X-ray crystal structure study for the cyclic tellurourea containing a benzimidazole group. The objective of this study was to elucidate the first crystal structure of such a cyclic tellurourea and compare the results to the corresponding analogues contain sulfur (İngeç *et al.*, 1999) and selenium (Aydın *et al.*, 1999; Akkurt *et al.*, 2004*a*).

The molecular structure of the title compound (I) is shown in Fig. 1. The molecule has a twofold screw axis through the midpoints of the C2—C2a and C4—C4a bonds and containing the atoms Te1 and C1 of the benzimidazole ring. The Te—C single bond length generally varies between 2.120 and 2.170 Å depending on the electron releasing effect of the ligand bonding to Te atom (Lappert *et al.*, 1980; Sadekov *et al.*, 1998; Närhi *et al.*, 2004; Singh *et al.*, 2006). In the title compound (I), the Te—C bond length [2.058 (4) Å] is short, which agrees with the results reported by Lappert *et al.* (1980). Tellurourea metal complexes may be described in terms of the resonance hybrids as shown in the scheme 2. For this reason, the Te?C bond may have partial double bond character.

All the geometric parameters of (I) are comparable with those in related compounds (Akkurt *et al.*, 2004*b*; Akkurt *et al.*, 2005; Türktekin *et al.*, 2004, Karaca *et al.*, 2005).

In the title molecule, the benzimidazole ring system (C1—C3/N1/C1a—C3a/N1a) is essentially planar, with maximum deviations of 0.007 (5) Å for C2 and -0.007 (5) Å for C2a. The symmetry-related phenyl rings (C8–C13 and C8a–C13*a*) are oriented at angles of 67.9 (4)° to the plane of the benzimidazole ring system.

### Experimental

A mixture of bis(1,3-di(3-phenylpropyl)benzimidazolidine-2-ylidene) (0.55 g; 0.78 mmol) and tellurium (0.22 g; 1.72 mmol) in toluene (10 ml) was heated under reflux for 2 h. The mixture was then filtered to remove unreacted tellurium and upon cooling the filtrate to 253 K, light yellow crystals of the title compound were obtained. (Yield: 0.54 g, 72%; m.p.: 390–391 K). ^1^H-NMR (CDCl_3_): δ 2.25 (q, 4H, CH_2_), 2.83 (t, 4H, CH_2_), 4.53 (t, 4H, N—CH_2_), 7.14–7.24 (m, 14H, Ar—H). ^13^C-NMR (CDCl_3_): δ 29.74, 32.99, 49.25, 110.26, 123.61, 126.20, 128.38, 128.50, 133.93, 140.73, 144.18. Analysis calculated for C_25_H_26_N_2_Te: C 62.29, H 5.40, N 5.82%. Found: C 62.52, H 5.50, N 5.82%.

### Refinement

The H atoms were placed in calculated positions and refined using a riding model with C—H in the range 0.93–0.97 Å and *U*_iso_(H) = 1.2*U*_eq_(C). Atoms C8, C9 and C13 in the phenyl ring appear to have unresolved disorder, so the distances C8—C9, C8—C13 and C9···C13 were restrained by *SHELXL DFIX* instructions [C8—C9 = 1.370 (12), C8—C13 = 1.336 (14) and C9···C13 = 2.229 (14) Å].

### Crystal data

**Table:**

C_25_H_26_N_2_Te	*Z* = 4
*M**_r_* = 482.08	*F*_000_ = 968
Tetragonal, *P*4_1_2_1_2	*D*_x_ = 1.394 Mg m^−^^3^
Hall symbol: P 4abw 2nw	Mo *K*α radiation λ = 0.71073 Å
*a* = 10.6004 (2) Å	Cell parameters from 55040 reflections
*b* = 10.6004 (2) Å	θ = 1.9–27.2º
*c* = 20.4365 (6) Å	µ = 1.31 mm^−^^1^
α = 90º	*T* = 296 K
β = 90º	Block, light yellow
γ = 90º	0.54 × 0.48 × 0.39 mm
*V* = 2296.42 (9) Å^3^	

### Data collection

**Table:**

Stoe IPDSII diffractometer	2264 independent reflections
Monochromator: plane graphite	2166 reflections with *I* > 2σ(*I*)
Detector resolution: 6.67 pixels mm^-1^	*R*_int_ = 0.049
*T* = 296 K	θ_max_ = 26.0º
ω scans	θ_min_ = 2.2º
Absorption correction: integration(X-RED32; Stoe & Cie, 2002)	*h* = −12→13
*T*_min_ = 0.539, *T*_max_ = 0.630	*k* = −13→13
28301 measured reflections	*l* = −25→25

### Refinement

**Table:**

Refinement on *F*^2^	Hydrogen site location: inferred from neighbouring sites
Least-squares matrix: full	H-atom parameters constrained
*R*[*F*^2^ > 2σ(*F*^2^)] = 0.037	*w* = 1/[σ^2^(*F*_o_^2^) + (0.0574*P*)^2^ + 1.6917*P*] where *P* = (*F*_o_^2^ + 2*F*_c_^2^)/3
*wR*(*F*^2^) = 0.105	(Δ/σ)_max_ < 0.001
*S* = 1.09	Δρ_max_ = 0.92 e Å^−^^3^
2264 reflections	Δρ_min_ = −0.48 e Å^−^^3^
99 parameters	Extinction correction: none
3 restraints	Absolute structure: Flack (1983), 889 Friedel pairs
Primary atom site location: structure-invariant direct methods	Flack parameter: 0.01 (6)
Secondary atom site location: difference Fourier map	

### Special details

**Table:**

Geometry. Bond distances, angles *etc*. have been calculated using the rounded fractional coordinates. All su's are estimated from the variances of the (full) variance-covariance matrix. The cell e.s.d.'s are taken into account in the estimation of distances, angles and torsion angles
Refinement. Refinement on *F*^2^ for ALL reflections except those flagged by the user for potential systematic errors. Weighted *R*-factors *wR* and all goodnesses of fit *S* are based on *F*^2^, conventional *R*-factors *R* are based on *F*, with *F* set to zero for negative *F*^2^. The observed criterion of *F*^2^ > σ(*F*^2^) is used only for calculating -*R*-factor-obs *etc*. and is not relevant to the choice of reflections for refinement. *R*-factors based on *F*^2^ are statistically about twice as large as those based on *F*, and *R*-factors based on ALL data will be even larger.

### Fractional atomic coordinates and isotropic or equivalent isotropic displacement parameters (Å^2^)

**Table:**

	*x*	*y*	*z*	*U*_iso_*/*U*_eq_	
Te1	0.13779 (3)	0.86221 (3)	0.75000	0.0633 (1)	
N1	0.3555 (4)	0.6989 (4)	0.79960 (17)	0.0585 (11)	
C1	0.2751 (4)	0.7249 (4)	0.75000	0.0550 (14)	
C2	0.4349 (5)	0.6006 (5)	0.7811 (2)	0.0653 (17)	
C3	0.5312 (6)	0.5400 (6)	0.8141 (3)	0.084 (2)	
C4	0.5917 (7)	0.4441 (7)	0.7828 (4)	0.101 (3)	
C5	0.3626 (6)	0.7656 (5)	0.8618 (2)	0.0630 (16)	
C6	0.4521 (5)	0.8761 (5)	0.8597 (2)	0.0677 (16)	
C7	0.4498 (6)	0.9471 (5)	0.9247 (3)	0.0723 (19)	
C8	0.5329 (9)	1.0605 (7)	0.9284 (5)	0.1219 (15)	
C9	0.4939 (9)	1.1694 (7)	0.9583 (5)	0.1219 (15)	
C10	0.5698 (9)	1.2703 (7)	0.9670 (5)	0.1219 (15)	
C11	0.6886 (9)	1.2679 (7)	0.9451 (5)	0.1219 (15)	
C12	0.7332 (10)	1.1639 (7)	0.9160 (5)	0.1219 (15)	
C13	0.6524 (9)	1.0587 (7)	0.9076 (5)	0.1219 (15)	
H3	0.55390	0.56380	0.85620	0.1020*	
H4	0.65670	0.40140	0.80390	0.1210*	
H5A	0.38990	0.70730	0.89550	0.0750*	
H5B	0.27910	0.79550	0.87340	0.0750*	
H6A	0.42790	0.93270	0.82460	0.0810*	
H6B	0.53690	0.84620	0.85100	0.0810*	
H7A	0.36370	0.97330	0.93340	0.0870*	
H7B	0.47420	0.88920	0.95920	0.0870*	
H9	0.41130	1.17400	0.97330	0.1460*	
H10	0.53940	1.34170	0.98830	0.1460*	
H11	0.74010	1.33830	0.95000	0.1460*	
H12	0.81630	1.16070	0.90150	0.1460*	
H13	0.68330	0.98660	0.88720	0.1460*	

### Atomic displacement parameters (Å^2^)

**Table:**

	*U*^11^	*U*^22^	*U*^33^	*U*^12^	*U*^13^	*U*^23^
Te1	0.0682 (2)	0.0682 (2)	0.0535 (2)	0.0114 (2)	0.0052 (2)	0.0052 (2)
N1	0.071 (2)	0.059 (2)	0.0454 (17)	−0.002 (2)	−0.0194 (17)	−0.0106 (15)
C1	0.061 (2)	0.061 (2)	0.043 (3)	−0.002 (2)	−0.004 (2)	−0.004 (2)
C2	0.073 (3)	0.061 (3)	0.062 (3)	0.003 (2)	−0.017 (2)	−0.017 (2)
C3	0.084 (4)	0.085 (4)	0.084 (3)	0.012 (3)	−0.039 (3)	−0.015 (3)
C4	0.082 (4)	0.085 (5)	0.136 (6)	0.024 (4)	−0.031 (4)	−0.020 (4)
C5	0.085 (3)	0.065 (3)	0.039 (2)	−0.001 (3)	−0.008 (2)	−0.0117 (18)
C6	0.085 (3)	0.064 (3)	0.054 (2)	−0.007 (3)	−0.014 (2)	−0.008 (2)
C7	0.091 (4)	0.062 (3)	0.064 (3)	0.005 (3)	−0.016 (3)	−0.021 (2)
C8	0.132 (3)	0.0736 (16)	0.160 (3)	−0.0065 (19)	−0.034 (3)	−0.0275 (19)
C9	0.132 (3)	0.0736 (16)	0.160 (3)	−0.0065 (19)	−0.034 (3)	−0.0275 (19)
C10	0.132 (3)	0.0736 (16)	0.160 (3)	−0.0065 (19)	−0.034 (3)	−0.0275 (19)
C11	0.132 (3)	0.0736 (16)	0.160 (3)	−0.0065 (19)	−0.034 (3)	−0.0275 (19)
C12	0.132 (3)	0.0736 (16)	0.160 (3)	−0.0065 (19)	−0.034 (3)	−0.0275 (19)
C13	0.132 (3)	0.0736 (16)	0.160 (3)	−0.0065 (19)	−0.034 (3)	−0.0275 (19)

### Geometric parameters (Å, °)

**Table:**

Te1—C1	2.058 (4)	C11—C12	1.339 (12)
N1—C1	1.353 (5)	C12—C13	1.417 (12)
N1—C2	1.392 (7)	C3—H3	0.9300
N1—C5	1.457 (6)	C4—H4	0.9300
C2—C3	1.382 (8)	C5—H5A	0.9700
C2—C2^i^	1.378 (6)	C5—H5B	0.9700
C3—C4	1.362 (10)	C6—H6A	0.9700
C4—C4^i^	1.444 (11)	C6—H6B	0.9700
C5—C6	1.508 (8)	C7—H7A	0.9700
C6—C7	1.527 (7)	C7—H7B	0.9700
C7—C8	1.492 (10)	C9—H9	0.9300
C8—C9	1.370 (12)	C10—H10	0.9300
C8—C13	1.336 (14)	C11—H11	0.9300
C9—C10	1.350 (12)	C12—H12	0.9300
C10—C11	1.337 (14)	C13—H13	0.9300
			
Te1···H5B	3.0200	H5A···H7B	2.4900
Te1···H3^ii^	3.1700	H5B···Te1	3.0200
Te1···H7B^ii^	3.2900	H5B···H7A	2.4200
Te1···H10^iii^	3.3200	H6A···H7B^ii^	2.5900
Te1···H3^iv^	3.1700	H6B···C13	2.8100
Te1···H7B^iv^	3.2900	H6B···H13	2.2700
Te1···H10^v^	3.3200	H7A···H5B	2.4200
Te1···H5B^i^	3.0200	H7A···H9	2.3300
C3···H5A	2.8600	H7A···C4^iv^	3.0900
C4···H7A^vi^	3.0900	H7A···H4^iv^	2.5800
C5···H3	2.9500	H7B···H5A	2.4900
C6···H13	2.7700	H7B···Te1^vii^	3.2900
C13···H6B	2.8100	H7B···H6A^vii^	2.5900
H3···C5	2.9500	H7B···Te1^vi^	3.2900
H3···H5A	2.4500	H9···H7A	2.3300
H3···Te1^vii^	3.1700	H10···Te1^viii^	3.3200
H3···Te1^vi^	3.1700	H10···Te1^ix^	3.3200
H4···H7A^vi^	2.5800	H13···C6	2.7700
H5A···C3	2.8600	H13···H6B	2.2700
H5A···H3	2.4500		
			
C1—N1—C2	109.3 (3)	N1—C5—H5A	109.00
C1—N1—C5	126.0 (4)	N1—C5—H5B	109.00
C2—N1—C5	124.7 (4)	C6—C5—H5A	109.00
Te1—C1—N1	126.1 (2)	C6—C5—H5B	109.00
Te1—C1—N1^i^	126.1 (2)	H5A—C5—H5B	108.00
N1—C1—N1^i^	107.8 (4)	C5—C6—H6A	110.00
N1—C2—C3	131.5 (4)	C5—C6—H6B	110.00
N1—C2—C2^i^	106.9 (4)	C7—C6—H6A	110.00
C2^i^—C2—C3	121.7 (5)	C7—C6—H6B	110.00
C2—C3—C4	117.8 (6)	H6A—C6—H6B	108.00
C3—C4—C4^i^	120.6 (7)	C6—C7—H7A	108.00
N1—C5—C6	112.6 (4)	C6—C7—H7B	108.00
C5—C6—C7	110.4 (4)	C8—C7—H7A	108.00
C6—C7—C8	115.6 (6)	C8—C7—H7B	108.00
C7—C8—C9	121.6 (8)	H7A—C7—H7B	107.00
C7—C8—C13	122.1 (7)	C8—C9—H9	118.00
C9—C8—C13	116.1 (8)	C10—C9—H9	118.00
C8—C9—C10	123.1 (9)	C9—C10—H10	120.00
C9—C10—C11	120.2 (8)	C11—C10—H10	120.00
C10—C11—C12	119.8 (8)	C10—C11—H11	120.00
C11—C12—C13	119.2 (9)	C12—C11—H11	120.00
C8—C13—C12	121.6 (8)	C11—C12—H12	120.00
C2—C3—H3	121.00	C13—C12—H12	120.00
C4—C3—H3	121.00	C8—C13—H13	119.00
C3—C4—H4	120.00	C12—C13—H13	119.00
C4^i^—C4—H4	120.00		
			
C2—N1—C1—Te1	−179.6 (3)	C2^i^—C2—C3—C4	0.8 (9)
C5—N1—C1—Te1	−1.9 (7)	C2—C3—C4—C4^i^	0.3 (10)
C2—N1—C1—N1^i^	0.4 (5)	C3—C4—C4^i^—C3^i^	−0.8 (11)
C5—N1—C1—N1^i^	178.1 (4)	N1—C5—C6—C7	176.6 (4)
C1—N1—C2—C3	−179.6 (6)	C5—C6—C7—C8	−178.9 (6)
C5—N1—C2—C3	2.7 (9)	C6—C7—C8—C9	139.2 (8)
C1—N1—C2—C2^i^	−1.1 (5)	C6—C7—C8—C13	−46.2 (11)
C5—N1—C2—C2^i^	−178.8 (5)	C7—C8—C9—C10	174.7 (9)
C1—N1—C5—C6	−89.3 (6)	C13—C8—C9—C10	−0.1 (15)
C2—N1—C5—C6	88.1 (6)	C7—C8—C13—C12	−175.0 (9)
N1—C2—C3—C4	179.1 (6)	C9—C8—C13—C12	−0.2 (15)
N1—C2—C2^i^—C3^i^	180.0 (5)	C8—C9—C10—C11	1.1 (16)
C3—C2—C2^i^—N1^i^	180.0 (5)	C9—C10—C11—C12	−1.7 (15)
C3—C2—C2^i^—C3^i^	−1.3 (8)	C10—C11—C12—C13	1.4 (15)
N1—C2—C2^i^—N1^i^	1.3 (6)	C11—C12—C13—C8	−0.4 (15)

Symmetry codes: (i) −*y*+1, −*x*+1, −*z*+3/2; (ii) *y*−1/2, −*x*+3/2, *z*−1/4; (iii) *y*−3/2, −*x*+3/2, *z*−1/4; (iv) *x*−1/2, −*y*+3/2, −*z*+7/4; (v) *x*−1/2, −*y*+5/2, −*z*+7/4; (vi) *x*+1/2, −*y*+3/2, −*z*+7/4; (vii) −*y*+3/2, *x*+1/2, *z*+1/4; (viii) −*y*+3/2, *x*+3/2, *z*+1/4; (ix) *x*+1/2, −*y*+5/2, −*z*+7/4.
